# Supplementary figures and images for: A unique cell division protein critical for the assembly of the bacterial divisome
Source: eLife. 2024 Oct 3;12:RP87922. doi: 10.7554/eLife.87922 (PMC11449484; doi:10.7554/eLife.87922)

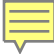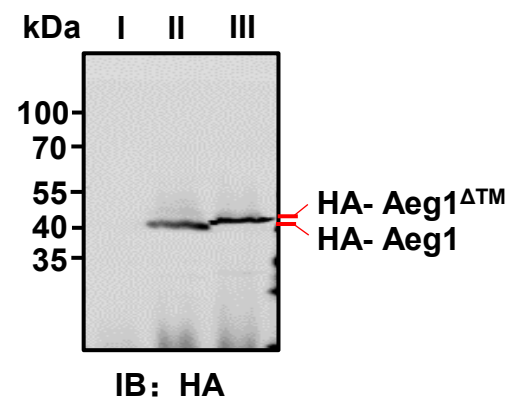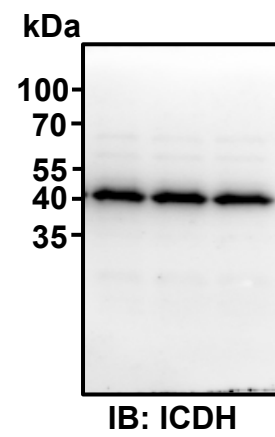

Supplement: Figure 1—source data 1. [file elife-87922-fig1-data1.zip › PDF file containing original western blots for Figure 1C.pdf]

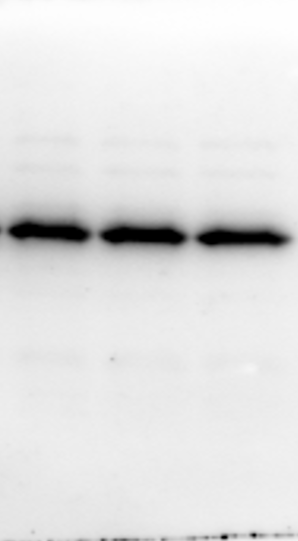

Supplement: Figure 1—source data 2. [file elife-87922-fig1-data2.zip › Raw unedited gels for figure 1C-lower panel.tif]

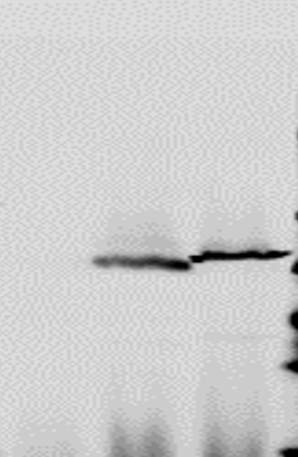

Supplement: Figure 1—source data 2. [file elife-87922-fig1-data2.zip › Raw unedited gels for figure 1C-upper panel.tif]

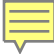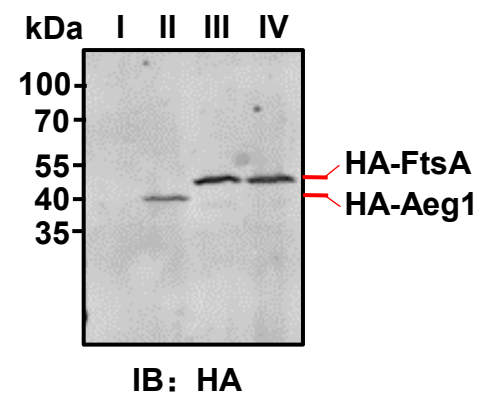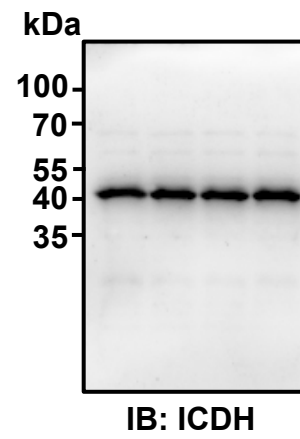

Supplement: Figure 3—source data 1. [file elife-87922-fig3-data1.zip › PDF file containing original western blots for Figure 3B.pdf]

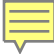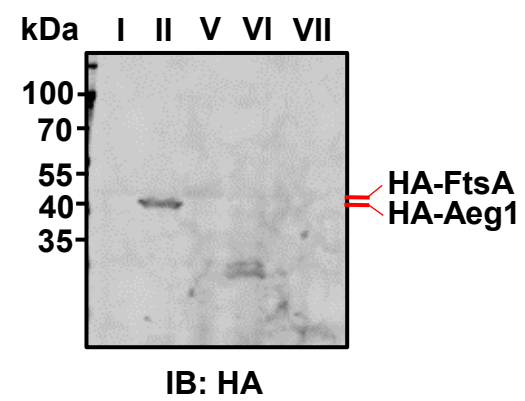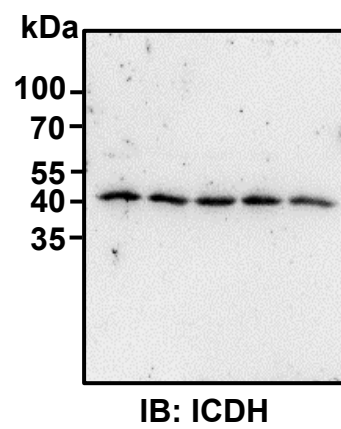

Supplement: Figure 3—source data 1. [file elife-87922-fig3-data1.zip › PDF file containing original western blots for Figure 3D.pdf]

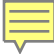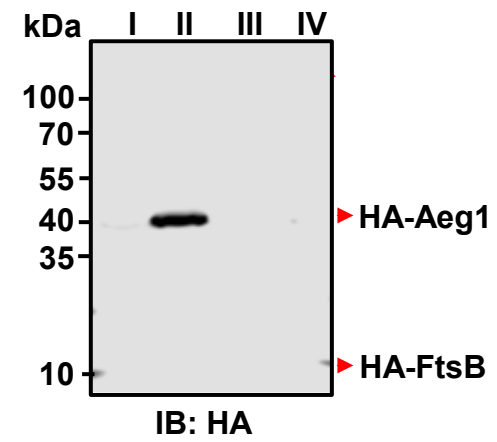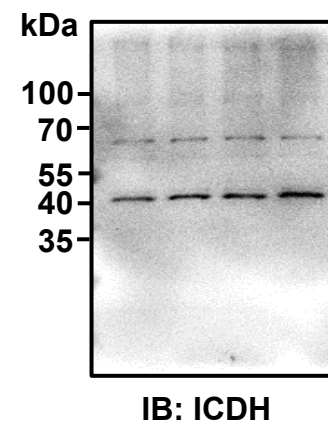

Supplement: Figure 5—source data 1. [file elife-87922-fig5-data1.zip › PDF file containing original western blots for Figure 5B.pdf]

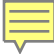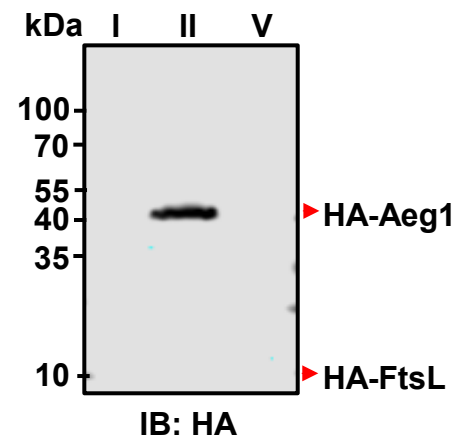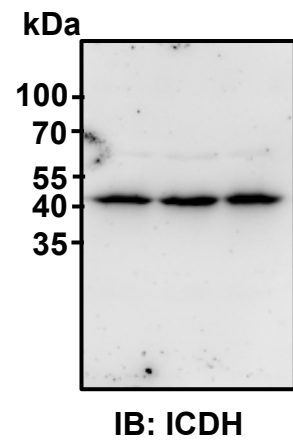

Supplement: Figure 5—source data 1. [file elife-87922-fig5-data1.zip › PDF file containing original western blots for Figure 5C.pdf]

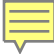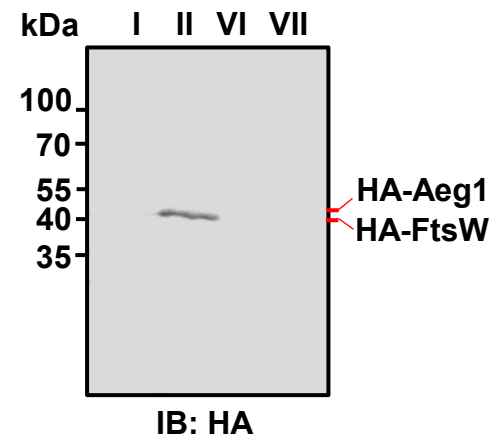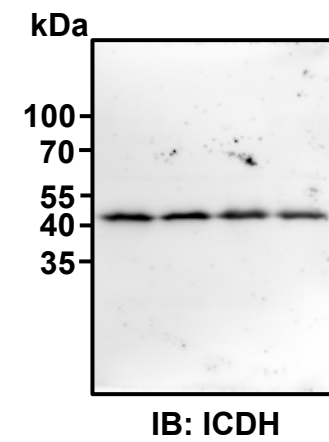

Supplement: Figure 5—source data 1. [file elife-87922-fig5-data1.zip › PDF file containing original western blots for Figure 5D.pdf]
