# Supplementary material for: A unique cell division protein critical for the assembly of the bacterial divisome
Source: eLife. 2024 Oct 3;12:RP87922. doi: 10.7554/eLife.87922 (PMC11449484; doi:10.7554/eLife.87922)
Supplement: Supplementary file 1. [file elife-87922-supp1.docx]

**Supplementary Files 1 candidate essential genes examined in this study**

| **Gene accession no.** | **Size (bp)** | **Gene function** |
| --- | --- | --- |
| A1S_0137 | 627 | hypothetical |
| A1S_1989 | 351 | hypothetical |
| A1S_1990 | 285 | hypothetical |
| A1S_2112 | 726 | hypothetical |
| A1S_2249 | 651 | hypothetical |
| A1S_2272 | 459 | hypothetical |
| A1S_2440 | 735 | hypothetical |
| A1S_2797 | 576 | hypothetical |
| A1S_3014 | 351 | hypothetical |
| A1S_3387 | 1290 | hypothetical |
