# Supplementary material for: A unique cell division protein critical for the assembly of the bacterial divisome
Source: eLife. 2024 Oct 3;12:RP87922. doi: 10.7554/eLife.87922 (PMC11449484; doi:10.7554/eLife.87922)
Supplement: Supplementary file 3. [file elife-87922-supp3.docx]

**Supplementary Files 3 Bacterial strains, plasmids and primers used in this study**

| **Strains or plasmids** | **Relevant features** | **Source** |
| --- | --- | --- |
| *A. baumannii* |  |  |
| ATCC 17978 | Type strain | (58) |
| 17978∆*A1S_0137* | Deletion mutant of the *A1S_0137* gene | This study |
| 17978∆*A1S_1989* | Deletion mutant of the *A1S_1989* gene | This study |
| 17978∆*A1S_1990* | Deletion mutant of the *A1S_1990* gene | This study |
| 17978∆*A1S_2112* | Deletion mutant of the *A1S_2112* gene | This study |
| 17978∆*A1S_2249* | Deletion mutant of the *A1S_2249* gene | This study |
| 17978∆*A1S_2272* | Deletion mutant of the *A1S_2272* gene | This study |
| 17978∆*A1S_2440* | Deletion mutant of the *A1S_2440* gene | This study |
| 17978∆*A1S_2797* | Deletion mutant of the *A1S_2797* gene | This study |
| 17978∆*A1S_3014* | Deletion mutant of the *A1S_3014* gene | This study |
| 17978∆*A1S_3387* | Deletion mutant of the *A1S_3387* gene | This study |
| *E. coli* |  |  |
| DH5α | *E. coli* strain for molecular cloning | (66) |
| BTH101 | *E. coli* reporter strains (a cya^-^deficient strain) | (38) |
| MT607 (pRK600) | Cm^R,^ *E. coli* helper strain for triparental conjugation, | (61) |
| Plasmids |  | (58) |
| pJL03 | Gm^R^, a pVRL1-based plasmid harboring P_BAD_ | (24) |
| pJL05 | Km^R^, a pDSK519-based plasmid harboring P_TAC_ | This study |
| pSR47s | Km^R^, sacB, a R6K plasmid for gene deletion | (25) |
| pJL03::A1S_0137 | Gm^R^, the *A1S_0137* gene cloned in pJL03 | This study |
| pJL03::A1S_1989 | Gm^R^, the *A1S_1989* gene cloned in pJL03 | This study |
| pJL03::A1S_1990 | Gm^R^, the *A1S_1990* gene cloned in pJL03 | This study |
| pJL03::A1S_2112 | Gm^R^, the *A1S_2112* gene cloned in pJL03 | This study |
| pJL03::A1S_2249 | Gm^R^, the *A1S_2249* gene cloned in pJL03 | This study |
| pJL03::A1S_2272 | Gm^R^, the *A1S_2272* gene cloned in pJL03 | This study |
| pJL03::A1S_2440 | Gm^R^, the *A1S_2440* gene cloned in pJL03 | This study |
| pJL03::A1S_2797 | Gm^R^, the *A1S_2797* gene cloned in pJL03 | This study |
| pJL03::A1S_3014 | Gm^R^, the *A1S_3014* gene cloned in pJL03 | This study |
| pJL03::Aeg1 | Gm^R^, the *A1S_3387* gene cloned in pJL03 | This study |
| pJL03::mCherry-Aeg1 | pJL03 expressing a mCherry::Aeg1 fusion | This study |
| pJL05::Aeg1 | Aeg1 cloned into pJL05 | This study |
| pJL05::Aeg1^ΔN^ | Aeg1^ΔN^ cloned into pJL05 | This study |
| pJL05::FtsA | FtsA cloned into pJL05 | This study |
| pJL05::FtsA_E202K_ | FtsA_E202K_ | This study |
| pJL05::FtsA_D124A_ | FtsA_D124A_ | This study |
| pJL05::FtsA_V144L_ | FtsA_V144L_ | This study |
| pJL05::FtsA_Q285W_ | FtsA_Q285W_ | This study |
| pJL05::FtsL_Q70K_ | :FtsL_Q70K_ | This study |
| pJL05::FtsB_E65A_ | FtsB_E65A_ | This study |
| pJL05::FtsB_D68H_ | FtsB_D68H_ | This study |
| pJL05::FtsW_M254I_ | FtsW_M254I_ | This study |
| pJL05::FtsW^M^_254I_ |  | This study |
| pJL05::FtsW_S274G_ | FtsW_S274G_ | This study |
| pJL05::eGFP-FtsZ | pJL05 expressing a FtsZ::eGFP fusion | This study |
| pJL05::eGFP-FtsA | pJL05 expressing a FtsA::eGFP fusion | This study |
| pJL05::eGFP-ZipA | pJL05 expressing a ZipA::eGFP fusion | This study |
| pJL05::eGFP-FtsK | pJL05 expressing a FtsK::eGFP fusion | This study |
| pJL05::eGFP-FsQ | pJL05 expressing a FtsQ::eGFP fusion | This study |
| pJL05::eGFP-FtsL | pJL05 expressing a FtsL::eGFP fusion | This study |
| pJL05::eGFP-FtsB | pJL05 expressing a FtsB::eGFP fusion | This study |
| pJL05::eGFP-FtsW | pJL05 expressing a FtsW::eGFP fusion | This study |
| pJL05::eGFP-FtsI | pJL05 expressing a FtsI::eGFP fusion | This study |
| pJL05::eGFP-FtsN | pJL05 expressing a FtsN::eGFP fusion | This study |
| pKT25 | T25 domain fusion for BTH | (67) |
| pUT18C-Flag | T18 domain fusion for BTH | This study |
| pKT25::Aeg1 | Aeg1 cloned into pKT25 | This study |
| pUT18C -Flag::FtsZ | FtsZ cloned into pUT18c-Flag | This study |
| pUT18C -Flag::FtsA | FtsA cloned into pUT18c-Flag | This study |
| pUT18C-Flag::ZipA | ZipA cloned into pUT18c-Flag | This study |
| pUT18C-Flag::FtsK | FtsK cloned into pUT18c-Flag | This study |
| pUT18C-Flag::FtsQ | FtsQ cloned into pUT18c-Flag | This study |
| pUT18C-Flag::FtsL | FtsL cloned into pUT18c-Flag | This study |
| pUT18C-Flag::FtsB | FtsB cloned into pUT18c-Flag | This study |
| pUT18C-Flag::FtsW | FtsW cloned into pUT18c-Flag | This study |
| pUT18C-Flag::FtsI | FtsI cloned into pUT18c-Flag | This study |
| pUT18C-Flag::FtsN | FtsN cloned into pUT18c-Flag | This study |

| **Primers** | **Sequence (Restriction enzyme sites are underlined)** | **Note** |
| --- | --- | --- |
| pCL1001 | ctgggatccgtgtttggcaaaacgcaa | *A1S_0137 5*F *BamH*I |
| pCL1002 | ctggtcgacttatttgcgtttaatcac | *A1S_0137* 3R *Sal*I |
| pCL1003 | ctggagctcaaacacttgatcaacgcg | *A1S_0137* up 5F *Sac*I KO |
| pCL1004 | ctgggatcccttgttaggtctttcagatacacc | *A1S_0137* up 3R *BamH*I KO |
| pCL1005 | ctgggatccttgcagcatagcggtattga | *A1S_0137* down 5F *BamH*I KO |
| pCL1006 | ctggtcgacgaaattgattttcataccaaccgag | *A1S_0137* down 3R *Sal*I KO |
| pCL1007 | ctggcggccgcgcaataggctcatgatacaactgac | *A1S_1989 5*F*Not*I |
| pCL1008 | ttcagggcccgctggttagattggcttggg | *A1S_1989* 3R *Apa*I |
| pCL1009 | aacggggcccgatactgaactcatccggtc | *A1S_1989* up 5F *Apa*I KO |
| pCL1010 | ctgggatcctgcacaaggtggtattgctgcaatt | *A1S_1989* up 3R *BamH*I KO |
| pCL1011 | ctgggatccgtgaaaactgtactcgtt | *A1S_1989* down 5F *BamH*I KO |
| pCL1012 | ctggtcgacctaccaatataagacatg | *A1S_1989* down 3R *Sal*I KO |
| pCL1013 | ctgggatccatgacgcaatcaactctttatc | *A1S_1990 5*F *BamH*I |
| pCL1014 | ctggtcgacttatttcaaagaaatgcatcgattaaatt | *A1S_1990* 3R *Sal*I |
| pCL1015 | ctggagctcgaacgtacataccagagatgtattc | *A1S_1990* up 5F *Sac*I KO |
| pCL1016 | ctgggatccatggtgataagaagcttgcac | *A1S_1990* up 3R *BamH*I KO |
| pCL1017 | ctgggatccgcggatctggttttacaatttaatc | *A1S_1990* down 5F *BamH*I KO |
| pCL1018 | ctgctcgagggccactcgctattgtgg | *A1S_1990* down 3R *Xho*I KO |
| pCL1019 | ctgggatccatggtattaatatttgctgc | *A1S_2112 5*F *BamH*I |
| pCL1020 | ctggtcgactcatactcgcaaagacaa | *A1S_2112* 3R *Sal*I |
| pCL1021 | ctggagctcagtcgagtctctaattgaaaaatacg | *A1S_2112* up 5F *Sac*I KO |
| pCL1022 | ctgggatccagaaaccagaacactgcac | *A1S_2112* up 3R *BamH*I KO |
| pCL1023 | ctgggatccatattttactcaaagattctccggc | *A1S_2112* down 5F *BamH*I KO |
| pCL1024 | ctggtcgacagcttactttttcgattaattggtattaattc | *A1S_2112* down 3R *Sal*I KO |
| pCL1025 | ctgggatccatgattgggcagcaaaga | *A1S_2249 5*F *BamH*I |
| pCL1026 | ctggtcgacctactcattgttatcttg | *A1S_2249* 3R *Sal*I |
| pCL1027 | ctggagctcttaaagaaacttcgaaatcgtcct | *A1S_2249* up 5F *Sac*I KO |
| pCL1028 | ctgggatccgtctattcctaaagttgccagtata | *A1S_2249* up 3R *BamH*I KO |
| pCL1029 | ctgggatcccttaagaaaagattgtggcaac | *A1S_2249* down 5F *BamH*I KO |
| pCL1030 | ctggtcgactgcaatgaagtgttattcatcctt | *A1S_2249* down 3R *Sal*I KO |
| pCL1031 | ctgggatccatgaaactcgcagaagca | *A1S_2272 5*F *BamH*I |
| pCL1032 | ctggtcgacttaattgattagatcaatt | *A1S_2272* 3R *Sal*I |
| pCL1033 | ctggcggccgcgcagtgaactgtcaccataac | *A1S_2272* up 5F *Not*I KO |
| pCL1034 | tcagggccctttttgttgattgcttcgaagtaaaag | *A1S_2272* up 3R *Apa*I KO |
| pCL1035 | acggggcccgtaaaaattcaggctgctaactg | *A1S_2272* down 5F *Apa*I KO |
| pCL1036 | ctgggatccatggctgaatggtaacttgaattt | *A1S_2272* down 3R *BamH*I KO |
| pCL1037 | ctgggatccatggtggagttaccctttc | *A1S_2440 5*F *BamH*I |
| pCL1038 | ctggtcgacttagataacttggtactg | *A1S_2440* 3R *Sal*I |
| pCL1039 | ctgctggcggccgctgcagattttattgcaacaggt | *A1S_2440* up 5F *Not*I KO |
| pCL1040 | tcagggcccgcgaacattcaaggcttgt | *A1S_2440* up 3R *Apa*I KO |
| pCL1041 | acggggccccaggatattcaagcacttgct | *A1S_2440* down 5F *Apa*I KO |
| pCL1042 | ctgggatcctcgtgtggctcaatttgagt | *A1S_2440* down 3R *BamH*I KO |
| pCL1043 | ctgggatccatgaccgcgaaaactttatatg | *A1S_2797 5*F *BamH*I |
| pCL1044 | ctggtcgacttataaagttttcggttcacctaca | *A1S_2797* 3R *Sal*I |
| pCL1045 | ctggagctcttgacagctttatttgttagccaac | *A1S_2797* up 5F *Sac*I KO |
| pCL1046 | ctgggatccaggaacaggctgaagcg | *A1S_2797* up 3R *BamH*I KO |
| pCL1047 | ctgggatcccgtaagcatttgaaagaaagtgtag | *A1S_2797* down 5F *BamH*I KO |
| pCL1048 | ctggtcgacatcatcttttttaggtaagttaactgttaaagtc | *A1S_2797* down 3R *Sal*I KO |
| pCL1049 | ctgggatccatggaatataaaggaagttgtca | *A1S_3014 5*F *BamH*I |
| pCL1050 | ctggtcgacttaaacagaacgcccatc | *A1S_3014* 3R *Sal*I |
| pCL1051 | ctgggatccgcgcgcttaactgcgagac | *A1S_3014* up 5F *Sac*I KO |
| pCL1052 | ctggaattcggcaaactttacctgaccacaatgaca | *A1S_3014* up 3R *EcoR*I KO |
| pCL1053 | ctggaattcttagaaaagataaaaataaattattttgatgggcgttctg | *A1S_3014* down 5F *EcoR*I KO |
| pCL1054 | ctggtcgacaccatgagcgagatatgcaccc | *A1S_3014* down 3R *Sal*I KO |
| pCL1055 | ctggagctctacaaaaagccgtttggtg | *A1S_3205* up 5F *Sac*I KO |
| pCL1056 | ctgggatccgaccactttattttctgtggtttc | *A1S_3205* up 3R *BamH*I KO |
| pCL1057 | ctgggatccacggtggtaatttctttacc | *A1S_3205* down 5F *BamH*I KO |
| pCL1058 | ctggtcgactcgatcgcaccacggtta | *A1S_3205* down 3R *Sal*I KO |
| pCL1059 | ctggagctcattacagcgcaaatggag | *A1S_0798* up 5F *Sac*I KO |
| pCL1060 | ctgggatccgataataatggcaacaacga | *A1S_0798* up 3R *BamH*I KO |
| pCL1061 | ctgggatcccgccatcatgtgattgat | *A1S_0798* down 5F *BamH*I KO |
| pCL1062 | ctgctcgagctgctgttgctgttttagg | *A1S_0798* down 3R *Xho*I KO |
| pCL1063 | ctggagctcaggtctgtttttggaaatc | *A1S_1896* up 5F *Sac*I KO |
| pCL1064 | ctgggatcctagcaatataagtttgcttga | *A1S_1896* up 3R *EcoR*I KO |
| pCL1065 | ctgggatccttggaaactaatgagacac | *A1S_1896* down 5F *EcoR*I KO |
| pCL1066 | ctggtcgacgagaggtaagagatttaggc | *A1S_1896* down 3R *Sal*I KO |
| pCL1067 | ctgggatccatgattgcgcctagacaagg | *aeg1* 5F *BamH*I |
| pCL1068 | ctggtcgacttaagatgctgcactactcgg | *aeg1* 3R *Sal*I |
| pCL1069 | ctggagctcacggaaactttgctggaa | *aeg1* up 5F *Sac*I KO |
| pCL1070 | ctgggatccaatgatcagtaggctagc | *aeg1* up 3R *BamH*I KO |
| pCL1071 | ctgggatcctcaattcctcaaaatgac | *aeg1* down 5F *BamH*I KO |
| pCL1072 | ctggtcgacaaaaatttctaaatctgcg | *aeg1* down 3R *Sal*I KO |
| pCL1073 | ctgggatccatggcctcatttgaatttatag | *ftsZ* 5F *BamH*I |
| pCL1074 | ctggtcgacttacttacgttgctgatttttca | *ftsZ* 3R *Sal*I |
| pCL1075 | ctgggatccatgagtgaagctgttccct | *ftsA* 5F *BamH*I |
| pCL1076 | ctggtcgacctaaaaaatggctttaagtttgc | *ftsA* 3R *Sal*I |
| pCL1077 | ctgggatccatggaaatcaatacgattattg | *zipA* 5F *BamH*I |
| pCL1078 | ctggtcgacctaggctgttgcctgtgctg | *zipA* 3R *Sal*I |
| pCL1079 | ctgggatccatgactgcggtgtcaagt | *ftsK* 5F *BamH*I |
| pCL1080 | ctgctcgagttaaactaaaatatcgcgctt | *ftsK* 3R *Xho*I |
| pCL1081 | ctgggatccatggcacaacttccggca | *ftsQ* 5F *BamH*I |
| pCL1082 | ctggtcgacttatggctttgcttttgtaccacct | *ftsQ* 3R *Sal*I |
| pCL1083 | ctgggatccatgaaaagcagtgatgaaatc | *ftsL* 5F *BamH*I |
| pCL1084 | ctggtcgacttacttattttgctctgaggt | *ftsL* 3R *Sal*I |
| pCL1085 | ctgggatccatgttagaagtatttcgttc | *ftsB* 5F *BamH*I |
| pCL1086 | ctggtcgacttaatctgggatgtctggtga | *ftsB* 3R *Sal*I |
| pCL1087 | ctgggatccatggcaggcttagctcag | *ftsW* 5F *BamH*I |
| pCL1088 | ctggtcgacttagaagtttgattcttccctctcag | *ftsW* 3R *Sal*I |
| pCL1089 | ctgggatccatggtagataagcgaacaaag | *ftsI* 5F *BamH*I |
| pCL1090 | ctggtcgacttacctgcgaataggattttc | *ftsI* 3R *Sal*I |
| pCL1091  pCL1092  pCL1093  pCL1094 | accatgcagcaatcgcat  catagctgtttcctgtgtg  acacaggaaacagctatgattgcgcctagacaaggg  cgattgctgcatggtagatgctgcactactcgg | pKT25-linearization*-*5F  pKT25-linearization*-*3R  Aeg1+T25-5F    Aeg1+T25-3R |
| pCL1095 | ctgggatccatgcaccagctcaatgaaaacc | *aeg1*^ΔN^ 5F *BamH*I |
| pCL1096 | ctgggtaccttaagatgctgcactactcgg | *aeg1* 3R *Kpn*I |
| pCL1097 | ctgggatccatggtgagcaagggcgag | *mCherry* 5F *BamH*I |
| pCL1098 | ctgagatctcttgtacagctcgtccatgc | *mCherry 3*R *Bgl*II |
| pCL1099 | ctgggatccatggtgagcaagggcg | *gfp* 5F *BamH*I |
| pCL1100 | ctgagatctcttgtacagctcgtccatgcc | *gfp 3*R *Bgl*II |
| pCL1101 | ccacgaccaaaagctattaacgcatttgataactgataacct | *ftsW*^M254I^ 5F |
| pCL1102 | aggttatcagttatcaaatgcgttaatagcttttggtcgtgg | *ftsW*^M254I^ 3R |
| pCL1103 | gtatgcgcttctggtaaatagccaagcttttgaacactatgccc | *ftsW*^S274G^ 5F |
| pCL1104 | gggcatagtgttcaaaagcttggctatttaccagaagcgcatac | *ftsW*^S274G^ 3R |
| pCL1105 | gtcgcaccaaatgtttgtttctcaatcaataaacgcccc | *ftsL*^Q70K^ 5F |
| pCL1106 | ggggcgtttattgattgagaaacaaacatttggtgcgac | *ftsL*^Q70K^ 3R |
| pCL1107 | gcactcgctagataataggcagaagtcacatggcttg | *ftsA*^D124A^ 5F |
| pCL1108 | caagccatgtgacttctgcctattatctagcgagtgc | *ftsA*^D124A^ 3R |
| pCL1109 | catattgattgggttttgaagccattcagatgagtcaccta | *ftsA*^V144L^ 5F |
| pCL1110 | taggtgactcatctgaatggcttcaaaacccaatcaatatg | *ftsA*^V144L^ 3R |
| pCL1111 | caactcaattcggctaatcgtccacggaccatcaataccctgtac | *ftsA*^Q285W^ 5F |
| pCL1112 | gtacagggtattgatggtccgtggacgattagccgaattgagttg | *ftsA*^Q285W^ 3R |
| pCL1113 | cattctttaggtcgaaaactgcagctgccaaaatacggttt | *ftsB*^E65A^ 5F |
| pCL1114 | aaaccgtattttggcagctgcagttttcgacctaaagaatg | *ftsB*^E65A^ 3R |
| pCL1115 | cgtaccattctttaggtggaaaacttcagctgccaa | *ftsB*^D68H^ 5F |
| pCL1116 | ttggcagctgaagttttccacctaaagaatggtacg | *ftsB*^D68H^ 3R |
| pCL1117 | ctgacatgtcgagaaaaggccatc | pJL05 5F *Pci*I |
| pCL1118 | cagggatccagcgtaatctggaacatcgtatgggtacatgggcagtcctcgg | pJL05 3R *BamH*I |
| pCL1119 | tcgagggactacaaagacgatgacgacaagggatccgatatcggtaccgagctcgaattcgtcgacat | pUT18C-Flag 5F |
| pCL1120 | cgatgtcgacgaattcgagctcggtaccgatatcggatcccttgtcgtcatcgtctttgtagtccc | pUT18C-Flag 3R |
